# Entomopathogenic nematodes targeting the control of the corn leafhopper, *Dalbulus maidis* (Hemiptera: Cicadellidae), under laboratory conditions

**DOI:** 10.2478/helm-2026-0003

**Published:** 2026-04-27

**Authors:** A. C. DE OLIVEIRA, V. ANDALÓ, M. L. DA MOTA, F. J. CARVALHO, L. S. DE FARIA, O. J. MARQUES

**Affiliations:** Federal University of Uberlândia, Institute of Agricultural Sciences, Monte Carmelo, MG, Brazil; Federal Institute of the Triângulo Mineiro, Department of Agronomy, Uberaba, MG, Brazil

**Keywords:** Biocontrol, Stunting, *Heterorhabditis*, *Steinernema*, Viruses, *Zea mays*

## Abstract

The corn leafhopper, *Dalbulus maidis* (DeLong & Wolcott) (Hemiptera: Cicadellidae), was considered a secondary pest in Brazilian corn crops. However, from 2015 onwards, there was a significant increase in its population and a high incidence of both pale and red wilt diseases. This species transmits these phytopathogens, elevating its status to a major agricultural pest. It has a high dispersal capability, significant reproductive potential, and adapts to adverse conditions, result in damage to crops by inhibiting plant growth and decreasing yield, leading to financial losses for farmers due to challenges in its control. Accordingly, the study aimed to evaluate the virulence of seven isolates of entomopathogenic nematodes belonging to the *Heterorhabditis* and *Steinernema* genera against adult *D. maidis*, as well as the lethal concentration (LC50), compatibility with insecticides and herbicides used in corn cultivation, and the nematode life cycle within the insect. There was no significant difference between the isolates, and they displayed a mortality rate exceeding 50%. The lethal concentration for *H. amazonensis* MC01 was 75 IJs adult^−1^, and for S. feltiae IBCB 47, it was 25 IJs adult^−1^. No phytosanitary product was found to be harmless to *H. amazonensis* MC01. The herbicide tembotrione did not affect the viability and infectivity of *S. feltiae* IBCB 47. The *H. amazonensis* MC01 and *S. feltiae* IBCB 47 isolates completed their life cycle in *D. maidis* adults, with the *S. feltiae* IBCB 47 isolate showing a longer life cycle than *H. amazonensis* MC01.

## Introduction

Corn (*Zea mays* L.) is one of the main commodities in Brazilian agribusiness. It is cultivated nationwide, and the 2021/2022 crop season production was 113.1 million tons, with a projected increase of 10.4 % for the 2022/2023 crop season. In Brazil, corn is cultivated intensively, allowing for up to three harvests a year: the first in spring/summer, the second in summer/fall/winter, and a third in certain Northeastern states (Conab, 2023; Von Pinho *et al*., 2017). However, intensive cultivation can increase pest populations and cause greater damage.

Pest attacks are a primary limitation to high yields. The corn leafhopper, *Dalbulus maidis* (DeLong & Wolcott) (Hemiptera: Cicadellidae), previously considered a secondary pest that caused sporadic damage, has been causing significant financial losses over the last few years. This pest is present throughout all phenological stages of the crop. However, the initial stages are most critical (Oliveira & Frizzas, 2022; Ramos *et al*., 2020; Waquil *et al*., 1998). The corn leafhopper can cause direct damage by sucking the photoassimilates and indirectly by transmitting the fine stripe virus and mollicutes responsible for pale and red wilt diseases (Oliveira & Frizzas, 2022). Mollicutes colonize the plant’s phloem, and the corn leafhopper can transmit them collectively or individually. The young and adult stages can acquire pathogens by feeding on sick plants, but only the adults transmit them to other plants (Bedendo, 2018). Infected females do not transmit the pathogens to their offspring but prefer laying eggs on infected plants. Both types of damage reduce the photosynthetic area (Ramos *et al*., 2020; Waquil *et al*., 1998).

Entomopathogenic nematodes of the *Heterorhabditis* and *Steinernema* genera are successful biological control agents that contain symbiotic bacteria from the *Photorhabdu*s and *Xenorhabdus* genera, respectively. The life cycle of EPNs is divided into eggs, 1st stage (J_1_) and 2nd stage (J_2_) juveniles, infective juveniles (IJ), 4th stage juveniles (J_4_), females, and males. The IJ enters the insect’s body through natural openings in the skin, such as spiracles, anus, and oral cavity. As soon as it enters the insect, it releases symbiotic bacteria into the hemolymph, causing the insect’s death from septicemia between 24 and 48 hours (Dolinski *et al*., 2017; Lacey & Georgis, 2012).

Many studies report the effectiveness of EPNs in controlling hemipterans, such as the brown stink bug, *Euschistus heros* (Fabricius) (Hemiptera: Pentatomidae) (Borges *et al*., 2023), and the green-belly stink bug, *Dichelops melacanthus* (Dallas) (Hemiptera: Pentatomidae) (Guide *et al*., 2019) and the spittlebug, *Mahanarva spectabilis* (Distant) (Hemiptera: Cercopidae) (Batista *et al*., 2014). This study aimed to assess the pathogenicity, virulence, and life cycles of *Heterorhabditis* and *Steinernema* species on *D. maidis* adults, and their compatibility with insecticides and herbicides used in corn cultivation.

## Materials and Methods

The corn leafhoppers were collected using a recyclable suction device in cornfields located in Monte Carmelo, MG, with the geographical coordinates 18°43’31.75”S, 47°31’ 32.06”W, at an altitude of 890 m. The breeding was maintained in a greenhouse using corn plants, SHS4070 hybrid from Santa Helena company, planted in pots with a five-liter capacity, and the plants were kept in pots until the V_5._ stage.

Thirty corn leafhoppers were added per plant and were covered with *voile* fabric. The pots received 15 grams of NPK fertilizer in the 04-14-08 formula and were watered every day as recommended for the crop.

### Selection of entomopathogenic nematodes

The nematodes were multiplied using *Tenebrio molitor* L. (Coleoptera: Tenebrionidae) larvae. The breeding of *T. molitor*followed the methodology of Potrich *et al*. (2007).

The experiment was carried out in gerbox containers measuring 11 x 11 x 3.5 cm, containing a piece of corn leaf measuring 10 x 11 cm and 1 % agar to keep the corn leaf turgid, allowing the corn leafhopper to feed. Ten adult corn leafhoppers were placed in each gerbox, with 6 repetitions per isolate, for a total of 60 insects per isolate. Isolates of *Heterorhabditis amazonensis* Andaló, Nguyen & Moino, represented by the isolates GL, MC01, UENP2 and UENP6, were used, as well as *Steinernema brazilense* Nguyen, Ginarte, Leite, dos Santos & Harakava (isolate IBCB06), *Steinernema carpocapsae* (Weiser) (isolate IBCB02), and *Steinernema feltiae* (Filipjev) (isolate IBCB47). These isolates were included to allow comparison of their ability to induce mortality in *D. maidis* under the same concentration conditions. Each isolate was tested separately, and distilled water served as the control, resulting in eight treatments. The isolates were inoculated onto adult *D. maidis* using an automatic pipette, with a concentration of 200 infective juveniles per insect in a volume of 0.5 ml in the gerbox type containers. Afterward, the gerboxs were sealed with cling film and kept under laboratory conditions. Insect mortality was observed to verify pathogenicity after 120 hours.

The adults were taken out of the gerboxs, kept in a dry room for 48 hours, and then dissected with a 1 % NaCl solution to confirm mortality brought on by the nematode. The presence of nematodes was observed under a stereoscopic microscope.

The collected data were subjected to variance analysis and were compared using the Scott-Knott test (p<0.05) with the assistance of the Exp.Des package in the R statistical software (Ferreira *et al*., 2021).

### Lethal concentration (LC50) of Heterorhabditis amazonensis MC01 and Steinernema feltiae IBCB47 isolates

To assess the lethal concentration caused by infective juveniles to *D. maidi*s adults, isolates *H. amazonensis* MC01 and *S. feltiae* IBCB47 were used, selected based on the results of the previous test. The experiments were carried out under the same conditions as the previous test.

The tested concentrations were 50, 100, 150, and 200 adult IJs per unit, and the control group only received distilled water. After 72 hours, insect mortality was assessed. The obtained data were analyzed using Probit in the R statistical software.

### Compatibility of H. amazonensis MC01 and S. feltiae IBCB47 with chemicals

The *H. amazonensis* MC01 and *S. feltiae* isolates were used to evaluate compatibility with registered phytosanitary products for corn cultivation. insecticides were tested: clorfluazuron, methomyl, chlorpyrifos, profenofos, and lufenuron, and four herbicides: atrazine, 2,4-D, glyphosate, and tembotrione. All the mentioned products are registered for corn cultivation by the Ministry of Agriculture, Livestock, and Supply (Agrofit, 2025).

The experiment followed the IOBC/WPRS protocol (Vainio, 1992) and involved nine treatments (products) and a control (distilled water). Each treatment was replicated five times. Double the recommended dose was used for the solution preparation, diluted with the nematode suspension. The volumes used to prepare 50 ml of each product solution were 0.06 ml of clorfluazuron, 1.25 ml of atrazine, 0.198 ml of methomyl, 0.60 ml of chlorpyrifos, 0.8 ml of profenofos + lufenuron, 0.2 ml of deltamethrin, 2 ml of glyphosate, 0.120 ml of tembotrione, and 0.35 ml of 2,4-D. The parameters evaluated were viability, infectivity, and production.

In a glass tube, 1 ml of the chemical solution and 1 ml of suspension containing 2,000 IJs were added, making a total volume of 2 ml with 1,000 IJs per mL, and the product was at the manufacturer-recommended dose. For the control, 1 ml of distilled water was added to 1 ml of the nematode suspension. The tubes were sealed with aluminum foil and kept under laboratory conditions.

Due to the pigmentation of some products, to ease the viewing of IJs under a stereo microscope, after the incubation phase (48 h), the tubes were cleaned to remove all chemical residues. The suspension was poured through a 500-mesh sieve to retain the nematodes and remove products. Subsequently, the retained nematodes were collected, and the volume was adjusted to 2 ml with distilled water.

Later, viability was assessed by taking 0.1 ml from each replication and counting live and dead juveniles. The count was done by adding the aliquot to the wells of an Elisa plate.

For infectivity, 3 ml of distilled water was added to the suspension. The tubes were kept in the fridge for 30 minutes. This procedure was performed three times to ensure complete removal of the chemical products. Subsequently, 1 ml of the suspension was taken and applied to *T. molitor* larvae placed on Petri dishes (9 cm in diameter) with two filter papers. The experiment was conducted under the described conditions and lasted five days. After this period, dead larvae were counted using symptom-based criteria specific to each tested species.

Dead larvae were transferred to White traps for production determination. The nematodes produced over five days were counted. The viability data of IJ and insect mortality were subjected to variance analysis, and nematode mortality values were adjusted using Abbott’s formula (1925).

The effect of chemical products on nematode infectivity (E) was calculated, classifying products as proposed by the International Organization for Biological and Integrated Control of Harmful Animals and Plants (IOBC): harmless (E <30 %), slightly harmful (E: 30 % – 79 %), moderately harmful (E: 80 % – 99 %), and harmful (E: >99 %).

### Life cycle of entomopathogenic nematodes in D. maidis

To assess the life cycle dynamics and reproductive output of *Heterorhabditis amazonensis* MC01 and *Steinernema feltiae* IBCB47 in *D. maidis* adults, we used concentrations of 5 IJ per adult (long cycle) and 100 IJ per adult (short cycle). Distilled water served as the control. Each treatment comprised 40 replicates, with one Petri dish (5 cm diameter) considered one replicate. Suspensions (0.5 ml per dish) were applied onto a corn leaf fragment (7 × 5 cm) placed on 1 % agar. One *D. maidis* adult was introduced per dish, totaling 40 insects per treatment.

Post-exposure, mortality was monitored, and evaluations of nematode penetration began 72 h after insect death. For penetration assessment, ten dead insects per treatment were randomly selected, transferred to a clean Petri dish (5 cm diameter) lined with dry filter paper, and held in the dark for 24 h. After this period, insects were washed with distilled water to remove IJs adhered to the cuticle and then dissected in 1 % NaCl. The number of IJs inside each insect and their developmental stage were recorded under a stereoscopic microscope. The remaining insects in each treatment continued to be observed to characterize the temporal progression of nematode development; the experiment lasted 264 h for *H. amazonensis* MC01 and 288 h for *S. feltiae* IBCB47.

## Results

### Selection of isolates of entomopathogenic nematodes

**Fig 1.**
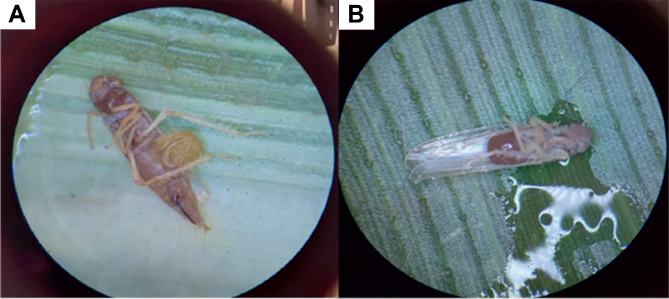
Adult *Dalbulus maidis* killed by entomopathogenic nematodes under laboratory conditions. A. *Heterorhabditis amazonensis* MC01. B. *Steinernema feltiae* IBCB47.

All tested isolates caused a mortality rate of over 50 % in *D. maidis* (Fig 1). There were no significant differences in the mortality percentages among the isolates (Table 1), indicating that all isolates are pathogenic to adult *D. maidis*.

**Table 1.** Mortality (%) of *Dalbulus maidis* adults caused by different isolates of entomopathogenic nematodes under laboratory conditions.

Treatment	Mortality (%)*
*Heterorhabditis amazonensis* GL	66.48 ± 4.20 a
*Heterorhabditis amazonensis* MC01	62.61 ± 13.71 a
*Heterorhabditis amazonensis* UENP2	78.68 ± 6.16 a
*Heterorhabditis amazonensis* UENP6	65.53 ± 7.29 a
*Steinernema brazilense* IBCB 06	52.85 ± 9.07 a
*Steinernema carpocapsae* IBCB 02	51.48 ± 6.64 a
*Steinernema feltiae* IBCB 47	77.62 ± 5.31 a
CV (%)	10.83

1 Mean ± Standard Error of the mean.

* Means followed by the same letter in the column do not differ by the Scott-Knott test at a 5 % probability level.

In subsequent steps, the *S. feltiae* IBCB47 isolates were selected as representatives of the *Steinernema* genus, and *H. amazonensis* MC01, a native isolate from the study region in Monte Carmelo, Minas Gerais, Brazil.

### Lethal concentration (LC50) of Heterorhabditis amazonensis MC01 and Steinernema feltiae IBCB47 isolates

**Fig. 2.**
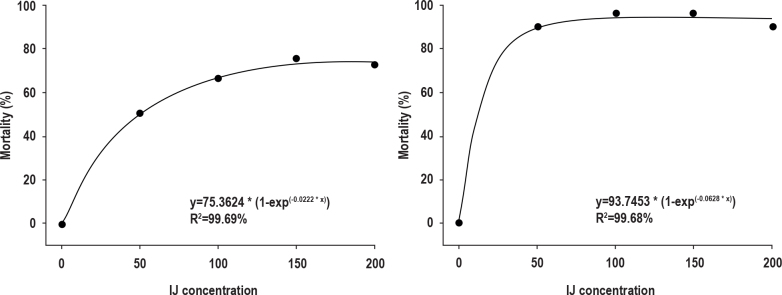
Lethal concentration of entomopathogenic required to kill *Dalbulus maidis* adults under laboratory conditions. A. *Heterorhabditis amazonensis* MC01. B. *Steinernema feltiae* IBCB47.

The exponential curve obtained by correlating different concentrations of the two isolates with the mortality of *D. maidis* was significant. The LC_50_ that results in adult mortality of *D. maidis* due to *H. amazonensis* MC01 was 75 IJ per adult and 173 IJ per adult for CL_75_ (Fig. 2A).

The LC_50_ causing adult mortality of *D. maidis* due to *S. feltiae* IBCB47 was 25 IJ per adult and 77 IJ per adult for CL_75_. However, even at higher concentrations, there was no increase in adult mortality (Fig. 2B).

### Compatibility of Heterorhabditis amazonensis MC01 and Steinernema feltiae IBCB47 isolates with phytosanitary products

All tested products affected viability and differed from the control treatment (Table 2). The infectivity potential of *H. amazonensis* MC01 to *T. molitor* larvae decreased after exposure to all products; the lowest infection rate was observed after exposure to the insecticide chlorpyrifos and the herbicide glyphosate. Insecticides methomyl and deltamethrin and the herbicide tembotrione did not significantly differ from the control treatment.

According to the IOBC protocol, none of the tested insecticides and herbicides were harmless to *H. amazonensis* MC01; only the insecticides chlorpyrifos and profenofos + lufenuron were classified as harmful. Herbicides atrazine, glyphosate, and tembotrione were classified as slightly harmful, and the herbicide 2,4-D as moderately harmful (Table 2).

**Table 2.** Compatibility of *Heterorhabditis amazonensis* MC01 with phytosanitary products after 48 hours of contact.

Treatment	Viability (%)*	Infectivity (%)*	MC	Rfin (%)	Rfec (%)	E (%)	IOBC Classification
Chlorfluazuron	68.85 ± 2.62c	35.00 ± 7.29b	24.28	51.07	2.33	77.68	Slightly Harmful
Atrazine	77.75 ± 2.03b	62.50 ± 12.50 a	14.53	30.36	0.00	44.88	Slightly Harmful
Methomyl	69.90 ± 1.87c	72.50 ± 9.18 a	23.16	13.92	19.56	56.64	Slightly Harmful
Chlorpyrifos	66.17 ± 3.63c	30.00 ± 8.48b	27.28	63.57	42.65	133.51	Harmful
Profenofos + Lufenuron	60.50 ± 3.27c	49.00 ± 9.00 b	33.52	50.00	13.36	130.59	Harmful
Deltamethrin	62.10 ± 4.53c	62.50 ± 12.50 a	31.73	24.52	37.30	93.55	Moderately Harmful
Glyphosate	77.54 ± 3.50 b	30.00 ± 10.23b	14.81	58.09	5.14	78.04	Slightly Harmful
Tembotrione	79.47 ± 2.71 b	70.00 ± 14.49 a	12.60	25.83	14.56	52.60	Slightly Harmful
2.4D	80.85 ± 2.90b	32.50 ± 12.25 b	11.12	57.62	11.09	79.83	Moderately Harmful
Control	90.97 ± 0.42 a	77.50 ± 8.29 a	0.00	0.00	0.00	0.00	Harmless
CV (%)	14.76	17.69					

1 Mean ± Standard Error of the mean.

* Means followed by the same letter in the column do not differ by the Scott-Knott test at a 5% probability level.

Regarding the *S. feltiae* isolate, the insecticides chlorfluazuron, chlorpyrifos, methomyl, and the herbicides 2,4-D, glyphosate, and tembotrione did not differ significantly from the control treatment (Table 3). The insecticide deltamethrin and the herbicide atrazine had the lowest viability rates. On the other hand, infectivity was reduced across all treatments, with the herbicide 2,4-D showing the lowest infectivity (60 %). Only the insecticide deltamethrin and the herbicide tembotrione did not significantly differ from the control treatment. Only the herbicide tembotrione was classified as harmless to *S. feltiae* IBCB47. The insecticides chlorfluazuron and chlorpyrifos, and the herbicide glyphosate, were classified as moderately harmful. However, the insecticides methomyl, profenofos + lufenuron, and deltamethrin, and the herbicides 2,4-D and atrazine, were classified as slightly harmful. None of the tested phytosanitary products were harmful to *S. feltiae* IBCB47.

**Table 3.** Compatibility of *Steinernema feltiae* IBCB 47 with phytosanitary products after 48 hours of contact.

Treatment	Viability (%)*	Infectivity (%)*	MC (%)	Rfin (%)	Rfec (%)	E (%)	IOBC Classification
Chlorfluazuron	92.71 ± 0.95 a	72.50 ± 9.18b	80.24	2.48	53.20	83.18	Moderately Harmful
Atrazine	88.43 ± 1.80c	72.50 ± 7.29b	6.88	27.50	0.00	34.38	Slightly Harmful
Methomyl	97.18 ± 0.72 a	65.00 ± 13.92b	0.00	35.00	43.17	75.93	Slightly Harmful
Chlorpyrifos	95.46± 0.67 a	80.00 ± 7.29b	0.00	20.00	67.55	87.07	Moderately Harmful
Profenofos + Lufenuron	91.74 ± 1.19b	65.00 ± 2.45b	3.48	35.00	0.20	38.67	Slightly Harmful
Deltamethrin	86.15 ± 2.81c	97.50 ± 2.50 a	9.42	2.50	37.81	49.73	Slightly Harmful
Glyphosate	96.44 ± 1.06 a	70.00 ± 10.89b	0.00	30.00	11.71	40.24	Moderately Harmful
Tembotrione	97.11 ± 1.26 a	87.50 ± 9.68 a	0.00	12.50	8.37	18.67	Harmless
2,4D	95.79 ± 1.39 a	60.00 ± 7.29b	0.00	40.00	7.82	47.04	Slightly Harmful
Control	95.07 ± 0.97 a	100.00 ± 0.00a	0.00	0.00	0.00	0.00	Harmless
CV (%)	3.38	24.24					

1 Mean ± Standard Error of the mean.

* Means followed by the same letter in the column do not differ by the Scott-Knott test at a 5% probability level.

### Life cycle of the Heterorhabditis amazonensis MC01 and Steinernema feltiae IBCB47 isolates in Dalbulus maidis

All development stages of the *H. amazonensis* MC01 isolate were observed in the short cycle (Table 4). In the long cycle, only the 2nd stage juvenile was not observed (Table 5).

**Table 4.** Duration of developmental stages of *Heterorhabditis amazonensis* MC01 in *Dalbulus maidis* at a concentration of 100 infective juveniles per insect (short cycle).

Developmental stage	Assessment time (h)							
	72	96	120	144	192	216	240	264
J_4_	0 - 10	10	18	3	10			
Hermaphrodites + eggs/J_1_				56				
Hermaphrodites + J_2_						18		
J_3_ and J_4_			50					
Females and Males				1	31			
Females + eggs/J_1_							279	755
Females + J_2_								
J_3_/JI				2				
J_4_ (2nd Generation)								

**Table 5.** Duration of developmental stages of *Heterorhabditis amazonensis* MC01 in *Dalbulus maidis* at a concentration of 5 infective juveniles per insect (long cycle).

Developmental stage	Assessment time (h)				
	72	144	168	216	264
J_4_	0 - 10	10	6		
Hermaphrodites + eggs/J_1_			3		
Hermaphrodites + J_2_					
J_3_ and J_4_					
Females and Males		1	31		
Females + eggs/J_1_				50	300
Females + J_2_					
J_3_/JI		2			
J_4_ (2nd Generation)					

In both periods, the *S. feltiae* IBCB47 isolate lasted longer compared to the *H. amazonensis* MC01 isolate (Tables 6 and 7). In the short cycle, the juvenile stage was observed 96 hours after application, while in the long cycle, it was 72 hours after application.

**Table 6.** Duration of developmental stages of *Steinernema feltiae* IBCB 47 in *Dalbulus maidis* at a concentration of 100 infective juveniles per insect (short cycle).

Developmental stage	Assessment time (h)							
	96	120	144	192	216	240	264	288
J_4_	92	68	137	4				
Hermaphrodites + eggs/J_1_	0 - 10	4	8		4			
Hermaphrodites + J_2_				17				
J_3_ and J_4_						15	6	
Females and Males						15	4	
Females + eggs/J_1_								
Females + J_2_								31
J_3_/JI							13	16
J_4_ (2nd Generation)								8

**Table 7.** Duration of developmental stages of *Steinernema feltiae* IBCB 47 in *Dalbulus maidis* at a concentration of 5 infective juveniles per insect (long cycle).

Developmental stage	Assessment time (h)						
	72	144	168	216	240	264	288
J_4_	0 - 10	3					
Hermaphrodites + eggs/J_1_	0 - 5	2	3				
Hermaphrodites + J_2_			3	3			
J_3_ and J_4_			1				
Females and Males					7		
Females + eggs/J_1_					8		
Females + J_2_							
J_3_/JI							9
J_4_ (2nd Generation)						2	4

## Discussion

All isolates in our screening caused mortality >50 % in *D. maidis* adults (Fig. 1), which supports the use of entomopathogenic nematodes against this pest and justified advancing *Heterorhabditis amazonensis* MC01 (native to Monte Carmelo, MG, Brazil) and *Steinernema feltiae* IBCB47 to subsequent assays (Table 1). High susceptibility of hemipterans to these agents is well documented: in *D. melachanthus* nymphs, Guide *et al*. (2019) reported >80 % mortality within 24 h for *H. amazonensis* GL (80.0 %), RSC05 (88.0 %), and *Steinernema* sp. IBCB-n27 (82.0 %). Consistently, Alves *et al*. (2009) found higher pathogenicity of *Heterorhabditis* spp. than *Steinernema* spp. against *Dysmicoccus texensis* (Tinsley) (Hemiptera: Pseudococcidae), and Zart *et al*. (2021) reported strong performance of *Heterorhabditis* on *D. brevipes*. Beyond the laboratory, Leite *et al*. (2005) documented ~70 % field efficacy of *Heterorhabditis* sp. CB-n5 against *Mahanarva fimbriolata* (Fabricius) (Hemiptera: Cercopidae), while Cecconello *et al*. (2022) showed high adult mortality of *E. heros* using *Heterorhabditis* IB-CB-n46 and NEPET 11 under laboratory and greenhouse conditions, together reinforcing that isolate identity shapes performance across Hemiptera and that certain *Heterorhabditis* isolates can perform under operational settings.

Dose–response analyses further characterized performance. For *H. amazonensis* MC01, LC_50_ = 75 IJ/adult and LC_75_ = 173 IJ/adult (Fig. 2A). For *S. feltiae* IBCB47, LC_50_ = 25 IJ/adult and LC_75_ = 77 IJ/adult (Fig. 2B). Increasing IBCB47 dose did not yield proportional mortality gains, suggesting a threshold response aligned with Batista *et al*. (2014), who reported that higher IJ densities did not improve performance of *H. amazonensis* RSC1 on *M. spectabilis*. Differences from Guide *et al*. (2019) using IBCB-n27 and from Nanzer *et al*. (2021) with *Steinernema diaprepesi* Nguyen & Duncan underscore host-isolate specificity and context dependence. Compatibility testing revealed distinct sensitivity profiles. For *H. amazonensis* MC01, all tested products reduced viability versus water (Table 2), with the lowest infection after the insecticide chlorpyrifos and the herbicide glyphosate; methomyl, deltamethrin, and tembotrione did not differ from the control for viability. Under IOBC categories, none of the products were harmless to MC01; chlorpyrifos and profenofos + lufenuron were harmful; atrazine, glyphosate, and tembotrione were slightly harmful; 2,4-D was moderately harmful. These patterns are consistent with Borges *et al*. (2023), who reported incompatibility of MC01 with chlorpyrifos, methomyl, and profenofos. For *S. feltiae* IBCB47 (Table 3), chlorfluazuron, chlorpyrifos, methomyl, 2,4-D, glyphosate, and tembotrione did not reduce viability relative to the control, whereas deltamethrin and atrazine produced the lowest viability. Infectivity declined under all products, with 2,4-D yielding the lowest infectivity (60 %); deltamethrin and tembotrione did not differ from the control for this endpoint. In broader context, Ozdemir *et al*. (2021) observed that diamide and spinosyn insecticides were not harmful to *S. feltiae*, while Amizadeh *et al*. (2019) reported high IJ mortality after exposure to abamectin and azadirachtin, highlighting that compatibility hinges on both isolate identity and active ingredient/formulation. Life-cycle observations aligned with these efficacy and compatibility patterns. *H. amazonensis* MC01 displayed all developmental stages in the short cycle (Table 4) and all except the second-stage juvenile in the long cycle (Table 5), indicating faster within-host progression. For *S. feltiae* IBCB47, juveniles were first recorded at 96 h (short cycle) and 72 h (long cycle) (Tables 6 and 7), indicating slower development. The influence of IJ density and host traits on cycle duration is consistent with foundational syntheses by Adams & Nguyen (2002), and with Grewal *et al*. (1994), who emphasized that developmental rate and recycling are determined by isolate pathogenicity, host size/quality, and environmental conditions. Moreover, reports of multiple generations of *H. amazonensis* RSC5 in *Galleria mellonella* L. (Lepidoptera: Pyralidae) (Andaló *et al*., 2009) corroborate our observation of robust within-host development for *Heterorhabditis* under favorable conditions.

Taken together, these results, anchored by our mortality screening (>50 % for all isolates), dose–response estimates for MC01 and IBCB47, compatibility profiles, and life-cycle timelines, indicate that careful matching of isolate, dose, and compatible chemistries is essential for integrating entomopathogenic nematodes into IPM against *D. maidis*. Future work should validate field performance across environments, refine dos–timing recommendations alongside compatible products, and quantify persistence and recycling in maize systems.

## Conclusion

All isolates tested were pathogenic to *D. maidis* adults. Under our conditions, the estimated lethal doses were LC_50_ = 22 IJ adult^−1^ for *S. feltiae* IBCB47 and LC_50_ = 75 IJ adult^−1^ for *H. amazonensis* MC01. Compatibility assays showed that the insecticides chlorpyrifos and profenofos + lufenuron were harmful to *H. amazonensis* MC01, whereas the herbicide tembotrione was harmless to *S. feltiae* IBCB47; nevertheless, both isolates exhibited reduced infectivity after exposure to several phytosanitary products, highlighting the need to manage product choice and application timing in IPM programs. Life-cycle assessments documented, for *H. amazonensis* MC01, the presence of all developmental stages in the short cycle and of all stages except the second-stage juvenile in the long cycle. For *S. feltiae* IBCB47, juveniles were first recorded at 96 h in the short cycle and at 72 h in the long cycle, and the developmental pace in both isolates depended on the number of infective juveniles applied.

## References

[j_helm-2026-0003_ref_001] Abbott W.S. (1925). A method of computing the effectiveness of an insecticide. J Econ Entomol.

[j_helm-2026-0003_ref_002] Adams B.J., Nguyen K.B., Gaugler R. (2002). Entomopathogenic Nematology.

[j_helm-2026-0003_ref_003] Agrofit - Sistemas de agrotóxicos fitossanitários [Phytosanitary pesticide systems] (2023).

[j_helm-2026-0003_ref_004] Alves V.S., Moino Junior A., Santa-Cecilia L.V.C., Andaló V., Souza G.C. (2009). Pathogenicity of entomopathogenic nematodes against the coffee root mealybug *Dysmicoccus texensis* (Tinsley) (Hemiptera: Pseudococcidae). Arq Inst Biol.

[j_helm-2026-0003_ref_005] Amizadeh M., Hejazi M.J., Niknam G., Askari-Saryazdi G. (2019). Interaction between the entomopathogenic nematode, *Steinernema feltiae* and selected chemical insecticides for management of the tomato leafminer, *Tuta absoluta*. BioControl.

[j_helm-2026-0003_ref_006] Andaló V., Moreira G.F., Cavalcanti R.S., Moino Junior A. (2009). Observations on the life cycle and pathogenicity of *Heterorhabditis amazonensis* (Rhabditida: Heterorhabditidae). Nematol Bras.

[j_helm-2026-0003_ref_007] Batista E.S.P., Auad A.M., Andaló V., Monteiro C.M.O. (2014). Virulence of entomopathogenic nematodes (Rhabditida: Steinernematidae, Heterorhabditidae) to spittlebug *Mahanarva spectabilis* (Hemiptera: Cercopidae). Arq Inst Biol.

[j_helm-2026-0003_ref_008] Bedendo I.P., Amorim L., Rezende J.A.M., Bergamin Filho A. (2018). Manual de fitopatologia: princípios e conceitos [Handbook of plant pathology: principles and concepts].

[j_helm-2026-0003_ref_009] Borges J.V.O., Andaló V., Temporim L.E., Arriero L.P.M., Lima L.M.R., Faria L.S.D. (2023). Virulence of entomopathogenic nematodes against neotropical brown stink bug (*Euschistus heros* [Fabricius], Hemiptera, Pentatomidae) and compatibility with phytosanitary products under laboratory conditions. J Agric Sci Technol.

[j_helm-2026-0003_ref_010] Cecconello D.M., Roggia S., Doneze G.S., Macedo M.F., Alves V.S. (2022). *Heterorhabditis amazonensis* to control *Euschistus heros* (Hemiptera: Pentatomidae) in laboratory and field conditions. Neotrop Entomol.

[j_helm-2026-0003_ref_011] Conab - Companhia Nacional de Abastecimento [National Supply Company] (2023). Acompanhamento da Safra Brasileira de Grãos: Safra 2022/23 7° levantamento abril 2023 [Monitoring the Brazilian Grain Crop: 2022/23 Crop 7th survey April 2023.

[j_helm-2026-0003_ref_012] Dash S.S., Koosari S., Ingole D.B., Kashyap D.P., Tambe V., Lavhe N. (2022). Compatibility studies of *Heterorhabditis indica* with newer insecticides under laboratory condition. Environ Conserv J.

[j_helm-2026-0003_ref_013] Dolinski C., Monteiro C., Andaló V., Leite L.G. (2017). Studies on entomopathogenic nematodes in Brazil: past and future. Nematoda.

[j_helm-2026-0003_ref_014] Fontes E.M.G., Valadares-inglis M.C. (2020). Controle Biológico de Pragas da Agricultura [Biological Control of Agricultural Pests].

[j_helm-2026-0003_ref_015] Guide B.A., Alves V.S., Fernandes T.A.P., Marcomini M.C., Meneghin A.M., Neves P.M.O.J. (2019). Pathogenicity and virulence of entomopathogenic nematodes against *Dichelops melacanthus* Dallas (Hemiptera: Pentatomidae). Semin Cienc Agrar.

[j_helm-2026-0003_ref_016] Lacey L.A., Georgis R. (2012). Entomopathogenic nematodes for control of insect pests above and below ground with comments on commercial production. J Nematol.

[j_helm-2026-0003_ref_017] Leite L.G., Machado L.A., Goulart R.M., Tavares F.M., Batista Filho A. (2005). Screening of entomopathogenic nematodes (Nemata: Rhabditida) and the efficiency of *Heterorhabditis* sp. against the sugarcane root spittlebug *Mahanarva fimbriolata* (Fabr.) (Hemiptera: Cercopidae). Neotrop Entomol.

[j_helm-2026-0003_ref_018] Magnabosco M.E.B., Andaló V., Faria L.S. (2019). Compatibility between entomopathogenic nematodes and crop protection products used in maize seed treatment. Semin Ciênc Agrár.

[j_helm-2026-0003_ref_019] Nanzer S.L.L., Recchia G.H., Chacon-Orozco J.G., Silva R.S.A., Cardoso J., Maringoli F., Leite L.G. (2021). Assessment of entomopathogenic nematodes and their symbiotic bacteria to control the stink bugs *Euschistus heros* and *Dichelops melacanthus* (Heteroptera: Pentatomidae) in the soybean-corn succession system. Turk J Zool.

[j_helm-2026-0003_ref_020] Oliveira C.M., Frizzas M.R. (2022). Eight decades of *Dalbulus maidis* (DeLong & Wolcott) (Hemiptera, Cicadellidae) in Brazil: what we know and what we need to know. Neotrop Entomol.

[j_helm-2026-0003_ref_021] Özdemir E., İnak E., Evlice E., Yüksel E., Delialioğlu R.A., Susurluk I.A. (2021). Effects of insecticides and synergistic chemicals on the efficacy of the entomopathogenic nematode *Steinernema feltiae* (Rhabditida: Steinernematidae) against *Leptinotarsa decemlineata* (Coleoptera: Chrysomelidae). Crop Prot.

[j_helm-2026-0003_ref_022] Potrich T.D., Lorini I., Voss M., Steffens M.C.S., Pavani D.P. (2007). Metodologia de criação de Tenebrio molitor em laboratório para obtenção de larvas [Methodology for rearing Tenebrio molitor in the laboratory to obtain larvae].

[j_helm-2026-0003_ref_023] Ramos A., Esteves M.B., Cortés M.T.B., Lopes J.R.S. (2020). Maize bushy stunt phytoplasma favors its spread by changing host preference of the insect vector. Insects.

[j_helm-2026-0003_ref_024] Vainio A. (1992). Guideline for laboratory testing of the side-effects of pesticides on entomophagous nematodes *Steinernema* spp. IOBC/WPRS Bulletin.

[j_helm-2026-0003_ref_025] Von Pinho R.G., Santo A.O., Von Pinho I.V., Galvão J.C.C., Borém A., Pimentel M.A. (2017). Milho: do plantio a colheita [Maize: from planting to harvest].

[j_helm-2026-0003_ref_026] Waquil J.M., Viana A.P., Cruz I., Santos P.J. (1998). Aspectos da biologia da cigarrinha-do-milho, *Dalbulus maidis* (DeLong & Wolcott) (Hemiptera: Cicadellidae) [Biologycal aspects of the corn leafhopper, *Dalbulus maidis* (DeLong & Wolcott) (Hemiptera: Cicadellidae)]. An Soc Entomol Bras.

[j_helm-2026-0003_ref_027] Zart M., Macedo M.F., Rando J.S.S., Doneze G.S., Brito C.P., Poletto R.S., Alves V.S. (2021). Performance of entomopathogenic nematodes on the mealybug, *Dysmicoccus brevipes* (Hemiptera: Pseudococcidae) and the compatibility of control agents with nematodes. J Nematol.

